# Do Vaccines Have a Role as a Cause of Autoimmune Neurological Syndromes?

**DOI:** 10.3389/fpubh.2020.00361

**Published:** 2020-07-28

**Authors:** Nicola Principi, Susanna Esposito

**Affiliations:** ^1^Università degli Studi di Milano, Milan, Italy; ^2^Pediatric Clinic, Department of Medicine and Surgery, Pietro Barilla Children's Hospital, University of Parma, Parma, Italy

**Keywords:** aluminum, guillain–barré syndrome, mercury, nervous system demyelinating syndrome, vaccine adverse events, vaccine autoimmunity

## Abstract

Vaccines are the most important preventive measure against infectious diseases presently available. Although they have led to the eradication or the elimination of some infectious diseases, concerns about safety are among the main reasons for vaccine hesitancy. In some cases, the biological plausibility of a given damage in association with the temporal association between vaccine administration and disease development makes it difficult to define causality and can justify hesitancy. Only well-conducted epidemiological studies with adequate evaluation of results can clarify whether a true association between vaccines and adverse event development truly exists. Autoimmune neurological syndromes that follow vaccine use are among these. In this narrative review, the potential association between vaccines and the development of these syndromes are discussed. Literature analysis showed that most of the associations between vaccines and nervous system autoimmune syndromes that have been reported as severe adverse events following immunization are no longer evidenced when well-conducted epidemiological studies are carried out. Although the rarity of autoimmune diseases makes it difficult to strictly exclude that, albeit exceptionally, some vaccines may induce an autoimmune neurological disease, no definitive demonstration of a potential role of vaccines in causing autoimmune neurological syndromes is presently available. Consequently, the fear of neurological autoimmune disease cannot limit the use of the most important preventive measure presently available against infectious diseases.

## Introduction

Vaccines are the most important preventive measure against infectious diseases presently available. They have led to the eradication of smallpox and the elimination of poliomyelitis in most countries. Moreover, their use has significantly reduced the development of several common and frequently severe infections that are associated with relevant morbidity and mortality and high costs for families and national health systems (1–3). Despite these undeniable advantages, vaccines are debated, vaccination coverage remains lower than desired and outbreaks of preventable diseases can occur (4). The recent increase in measles incidence rates in many countries around the world is the best example in this regard (5).

Concerns about safety are among the main reasons for vaccine hesitancy (6). In most cases, particularly when healthy children are considered, concerns about safety have no scientific basis and derive from myths or poor knowledge (7). In some other cases, the biological plausibility of a given damage in association with the temporal association between vaccine administration and disease development makes it difficult to define causality and can justify hesitancy (8). Only well-conducted epidemiological studies with adequate evaluation of results can clarify whether a true association between vaccines and adverse event development truly exists (9). Autoimmune neurological syndromes that follow vaccine use are among these.

In this narrative review, the potential association between vaccines and the development of these syndromes will be discussed. Data for discussion have been derived from studies published in English and reported in PubMed and Scopus from January 1, 1985 to February 29, 2020, using “vaccine hesitancy,” “vaccine adverse events,” “vaccine autoimmunity,” “nervous system demyelinating syndrome,” “Guillain–Barré syndrome,” “aluminum,” and “mercury” as key words.

## Vaccines and Central Nervous System (CNS) Demyelinating Syndromes

Central nervous system (CNS) demyelinating syndromes include acute disseminated encephalomyelitis (ADEM), transverse myelitis (TM), neuromyelitis optica (ON), and multiple sclerosis (MS) (10). In general, ADEM is a monophasic disease that occurs mainly in children, whereas MS is a chronic disease of young adults characterized by an alternation of relapses and remissions. However, as ADEM can recur and both TM and ON can be associated with ADEM or MS, distinction between these conditions can be difficult, and a clinical continuum has been supposed. Although typical cases have relevant differences in some clinical manifestations and laboratory test results, all these diseases are thought to be due to an overactive or dysfunctional immune response to self-antigens (11).

Mechanisms that lead to autoimmunity are unknown, although it is supposed that viral and, more rarely, bacterial infections play a fundamental role in triggering abnormal immune responses. Most cases are preceded by an infectious disease, with *Chlamydia pneumoniae*, herpesvirus (6), Epstein–Barr virus, and endogenous retroviruses being the most frequently causative agents (12). Molecular mimicry, or the fulminant activation of lymphocytes by microbial superantigens, are the most commonly proposed mechanisms for demyelinating syndrome development (13, 14).

For similarity with what has been shown for infections, it was thought that vaccines could also lead to demyelination. In some cases, a strict temporal relationship between vaccine administration and the development of CNS demyelinating syndromes has been reported, favoring the hypothesis that vaccine administration, despite being able to ensure protection against some infections, could be deleterious and cause clinical problems sometimes even more severe than the prevented disease (15). Most of the reported cases concerned hepatitis B vaccine (HBV) and human papillomavirus vaccine (HPV). The potential role of HBV was first speculated in 1997 in France after the evidence that a large HBV campaign involving neonates, children and young adults at risk had been followed by a sudden and unexpected increase in the number of CNS demyelinating syndromes, including MS, within 8 weeks of HBV administration (16). This resulted in a complete suspension of the national vaccination programme and stimulated the activation of several studies to confirm or refute the association. In most cases, no significant increase in demyelinating syndrome onset within months or years of HBV administration was demonstrated, regardless of the type of vaccine used. Moreover, it was shown that HBV did not cause the exacerbation of previously evidenced demyelinating conditions (17–21). On the other hand, when a potential relationship was found, the application of Hill's criteria of causation to the data collected with these studies led to the conclusion that the correlation between HBV and MS might be causal (22). Similar results were reported when the impact of HPV on CNS demyelination was evaluated, regardless of the preparation, bivalent, or quadrivalent, used. A systematic review of 5 observational studies, nine reviews, and one randomized clinical trial (RCT) confirmed no significant association between HPV and MS. The risk of MS development following vaccination varied from 1.54 [95% confidence interval [CI], 0.04–8.59] to 1.37 (95% CI, 0.74–3.20). An evaluation of the case-control studies revealed an odds ratio (OR) ranging from 0.3 (95% CI, 0.1–0.9) to 1.60 (95% CI, 0.79–3.25) without significant differences between groups (23). A more recent systematic review and meta-analysis of 11 pharmacoepidemiological studies including a control group with non-significant heterogeneity confirmed that no significant association between HPV and CNS demyelination could be demonstrated (OR 0.96; 95% CI, 0.77–1.20). Similar results were found when MS and ON were considered separately. Sensitivity analyses did not modify conclusions (24). The safety of HPV was further demonstrated in a recent review and meta-analysis of post-licensure observational studies. The OR for MS was 0.96 (95% CI, 0.77–1.21), and for all the other CNS demyelinating syndromes it was 1.02 (95% CI, 0.77–1.33) (25).

An analysis of the impact of other vaccines on MS or other CNS demyelination syndromes is also reassuring, although in many cases available data were collected with studies showing some methodological limitations. Frequently, very few vaccinated subjects were enrolled, time intervals from immunization and symptom onset were not precisely defined, and potentially confounding factors were not correctly considered (26, 27). A good example in this regard is given by a case-centered analysis of the potential association of vaccine administration and TM and ADEM development. This study was carried out with the data collected by the US Vaccine Safety Datalink and regarded the follow-up of 64 million vaccine doses in the 5–28 days after immunization, during which 7 and 8 cases of TM and ADEM, respectively, occurred. TM was not associated with any of the administered vaccines. The same was true for ADEM, except for the Tdap (adolescent and adult tetanus, reduced diphtheria, acellular pertussis) vaccine. However, the association was based on only 2 exposed cases, leading to an OR of 15.8 (95% CI, 1.2–471.6; *p* = 0.04). The calculated excess risk was 0.385 cases (95% CI, −0.04 to 1.16) per million doses. The authors themselves highlighted that the number of disease cases was too small to draw firm conclusions and that the results could be due to chance alone (28). However, a systematic review of 51 published studies regarding immunization and MS showed that the use of HBV, HPV, seasonal influenza, measles-mumps-rubella (MMR), varicella, tetanus, BCG, polio, and diphtheria vaccines did not cause any appreciable increase in the risk of MS development or relapse (29). Interestingly, in another study, when any type of vaccination was found to be associated with an increased risk of CNS demyelinating syndromes within 30 days after vaccination in subjects <50 years of age, it was evidenced that in some of these subjects, a previous risk factor for demyelinating syndrome development was already present. Starting from this finding and the evidence that in the same study none of the vaccines was associated with any demyelinating syndrome 30 days after vaccination, the authors concluded that vaccines were not the cause of disease. It was assumed that the vaccines could have acted as a proinflammatory cofactor in subjects with subclinical autoimmunity, showing a condition that would have spontaneously emerged in later times (30).

However, the need for a meticulous evaluation of epidemiological studies seems clearly shown by the results of a nested case-control study carried out in China with the aim of analyzing the potential relationship between vaccines and ADEM. The cases of patients with this disease hospitalized during the period 2011–2015 after the introduction of the Expanded Program of Immunization in that country were evaluated (31). A total of 272 patients with ADEM and 1,096 controls were enrolled. No association with ADEM was demonstrated for HBV, hepatitis A, influenza, live polio, diphtheria, acellular pertussis, tetanus, MMR, varicella, or Japanese encephalitis vaccination within the 180 days after vaccination regardless of the age of the enrolled individuals. However, further analyses revealed that the immunization of children was associated with a statistically significant increase in the risk of ADEM in the 31–60-days exposure interval (OR 4.04; 95% CI, 1.07–12.96), although this risk was not evident for the 0–30 and 61–180-days intervals. The increased risk was not specific to a vaccine and was not evidenced among adults in any of the study subperiods. Moreover, when pediatric and adult data were pooled, the increased risk shown in the 31–60 days after immunization was no longer evincible. Reasons for this finding are not precisely defined. However, as children receiving vaccines were more likely to have had an infectious disease, mainly a respiratory tract infection, in the 6 months before symptom onset (*p* < 0.05), it could be supposed that infections could have triggered ADEM and that this was already active when vaccines were given.

In conclusion, no epidemiological study carried out with undisputable methods has clearly shown that vaccines can cause CNS demyelinating syndromes. Fear for the development of these syndromes cannot be considered a reason for the limitation of vaccine use.

## Guillain–Barre' Syndrome (GBS)

Guillain–Barré syndrome (GBS) is an acute polyradiculoneuropathy that presently is the most common cause of acute flaccid paralysis worldwide. It annually occurs in 0.4–4.0 individuals per 100,000 population, mainly in males older than 75 years of age (32). In most cases, GBS follows an infection due to *Campylobacter jejuni* or cytomegalovirus and, more rarely, *Mycoplasma pneumoniae*, influenza virus, Zika virus, and Epstein–Barr virus (33–37). The association of GBS with other pathogens, such as measles virus, varicella-zoster virus and *Haemophilus influenzae*, is debated (38–40). Four main subtypes of GBS, which are differentiated by nerve electrophysiological findings, have been identified: acute inflammatory demyelinating polyradiculoneuropathy (AIDP), acute motor axonal neuropathy (AMAN), acute motor and sensory axonal neuropathy (AMSAN), and Miller Fisher syndrome (41). AIDP is the most common. For all the subtypes, an autoimmune pathogenesis has been supposed and, in some cases, clearly evidenced. In AIDP, the evidence of high T cell concentrations in damaged nerves and myelin proteins in the sera of patients supports the hypothesis that lesions can be due to combined cell-mediated and humoral immune reactions (42). Alternatively, it was suggested that autoantibodies against cell adhesion proteins localized at Ranvier's nodes were possible targets in AIDP (43). In contrast, in AMAN and AMSAN, where no demyelination occurs and lesions affect only nerve axons, the damage depends on the presence of IgG antibodies against gangliosides. In this case, molecular mimicry seems to be the most important factor for autoimmunity development. The structure of several gangliosides resembles the structure of bacterial components, as clearly shown by the evidence that the lipopolysaccharides of the outer membrane of *Campylobacter jejuni* have molecular compositions quite similar to those of GM1 and GD1a gangliosides of nerve axons (44, 45). On the other hand, GBS can be reproduced by immunizing experimental animals with gangliosides (46, 47) or with *Campylobacter jejuni* lipopolysaccharide from patients with GBS (48).

The role of vaccines in conditioning GBS has been debated for several years, especially when it was found that inactivated nervous tissue anti-rabies vaccine administration could be associated with the development of GBS (49). However, epidemiological studies have excluded any role of polio and diphtheria-tetanus-pertussis vaccines in the determination of GBS. The same seems true for pneumococcal, varicella, hepatitis A and B and *Haemophilus influenzae* type b vaccines (50). Doubts are still raised for measles and MMR vaccines, HPV, quadrivalent meningococcal vaccine (MCV4), and influenza vaccine. Regarding the measles vaccine and the MMR vaccine, no definitive conclusion can be drawn, although pharmaceutical companies that produce and market these vaccines report this potential risk in the product information leaflets (51). The results of the studies specifically planned to evaluate the potential association between measles-virus-containing vaccines and GBS development are conflicting. However, most of the data showing an increased occurrence of GBS in subjects immunized with these vaccines have significant methodological limitations that preclude any valid conclusion. For example, in many individuals, the MMR vaccine was given together with other vaccines, making it impossible to state the true importance of the MMR vaccine as a cause of disease (52–56). This explains why the US Institute of Medicine stated that available data do not allow us to establish whether there is an association between the MMR vaccine and GBS (57). On the other hand, if a real risk exists, it must be extremely low, as risk could not be demonstrated in several studies, enrolling a very great number of subjects, that have measured and compared the incidence of GBS in periods with different MMR vaccination coverages. Evaluations carried out in Finland (58), Iran (59), and South America (60) have shown that the incidence of GBS was quite similar in periods with and without MMR vaccine use. Given these findings and the dramatic benefits of the MMR vaccine, the risk of GBS cannot be considered a limitation for MMR vaccine use.

An apparent association between GBS and HPV administration was evidenced in a study carried out in France, in which it was found that the development of this polyradiculoneuropathy within 33 months of vaccination was several times more frequent than in unvaccinated controls (1.4 vs. 0.4 per 100,000 cases) (61). This finding led the World Health Organization Global Advisory Committee on Vaccine Safety to conclude that, despite being low, the risk was high enough to recommend further studies in adequately sized populations (62). However, several subsequent retrospective studies carried out on populations that had received millions of HPV doses have yielded completely opposite results (63, 64). Neither bivalent nor tetravalent HPV could be associated with an increased risk of GBS development, as no statistically significant difference between vaccinated and control groups in the incidence of GBS could be demonstrated. The lack of any evidence of a causative role of HPV was further confirmed by passive surveillance reports (65) and active monitoring by both the US Vaccine Safety Datalink (66) and the US Vaccine Adverse Events Reporting System (67). All these findings led the World Health Organization Global Advisory Committee on Vaccine Safety to change its statement. It was reported that a true relationship between GBS and HPV could not be demonstrated, and even if existing, statistical calculations allow us to conclude that it could not be >1 case per million doses (68). For the MMR vaccine, no limitation in the use of HPV can be derived from the hypothetical risk of GBS development.

The potential association between the tetravalent meningococcal (groups A, C, Y and W-135) polysaccharide diphtheria toxoid conjugate vaccine (MCV4) and GBS was supposed when, a few months after the inclusion of this vaccine in the US immunization schedule, five GBS cases were diagnosed among adolescents who had been immunized 2–4 weeks before (69). Although this finding was not considered a limitation for MCV4 use by the US Advisory Committee of Immunization Practice (ACIP) (70), the US Food and Drug Administration recommended that this vaccine was not used in subjects with a previous history of GBS and that further adequate studies to evaluate the problem should be performed (71). In response to these demands, several epidemiological evaluations, in some cases involving millions of MCV4 doses, were conducted (72–74). None of them showed any significant correlation between the vaccine and GBS, as the risk of GBS development after MCV4 administration was not different from that usually reported in unvaccinated subjects. It was highlighted that statistical calculations had shown that, even considering the less favorable circumstances, <1.5 GBS excess cases per million vaccinations could occur after GBS use. These findings were considered fully satisfactory and led the ACIP to remove the limitations to the use of MCV4 and any other tetravalent conjugate meningococcal vaccine regardless of conjugation protein characteristics (75).

Regarding influenza vaccine, a direct relationship of a strict linkage between this vaccine and GBS development was first supposed in 1976 when the receipt of a swine influenza vaccine in the USA was associated with a significantly greater than expected incidence of GBS (76). It was calculated that the influenza vaccine could cause one additional case of GBS every 100,000 doses (77). In the following years, a great number of studies attempted to verify whether this association truly existed (78–84). The results were conflicting. In most of the cases, no relationship was evidenced, but two well-conducted studies carried out in Canada (82) and in the USA (84) showed that in the 6 weeks after immunization, the risk of GBS development was significantly increased (relative risk [RR] 1.45; 95% CI, 1.05–1.99; *p* = 0.02 and 1.7; 95% CI, 1.0–2.8; *p* = 0.04, respectively). Conflicting results were also obtained from studies carried out during the 2009 influenza pandemic. However, in this case, most of the studies revealed a slight but significant increase in the risk of GBS development in immunized subjects, independent of the type of vaccine used and the geographic area where the study was carried out. In the USA, where only non-adjuvanted vaccines were administered, the relative risk of GBS was 2.35 (95% CI, 1.4–4.0), with 1–3 additional cases per million persons vaccinated (85). Similar data were obtained in Canada and Europe (86–90). Moreover, an international study carried out in 15 countries where both adjuvanted and non-adjuvanted pandemic vaccines were used, the calculated RR was 2.42 (95% CI, 1.58–3.72) (91). However, some of the studies showing that influenza vaccines could be a potential risk for GBS development were strongly criticized because of significant methodological problems, making the results difficult to interpret. Case series were considered inadequate to establish causality. Moreover, in uncontrolled observational studies carried out over time, confounding factors such as changing case definitions or improving case identification could have affected the apparent incidence and prevalence of the adverse outcome. Consequently, the potential relationship between influenza vaccines and GBS remains an unsolved problem. However, if influenza vaccines can cause GBS, the risk is very low and certainly lower than that due to influenza disease. There are data that indicate that the relative risk of GBS in the 6 weeks after an episode of influenza is significantly greater (RR 15.81; 95% CI, 10.28–24.32) than that ascribed to vaccination even in the most pessimistic assessments (92). This seems to indicate that the influenza vaccine remains a fundamental protective measure and should not be feared to cause GBS. However, a means to reduce the risk of GBS intrinsically related to influenza virus infection must be considered.

## Aluminum-Related Neurological Syndromes

Aluminum (Al) is added to several vaccine preparations (tetanus, hepatitis A, hepatitis B, human papillomavirus, *Haemophilus influenzae* type b, and infections due to *Streptococcus pneumoniae* and *Neisseria meningitidis*) to increase the immune response to vaccine antigens and improve the protection evoked by antigens alone (93). It stimulates both the innate and adaptive immune systems through the activation of antigen-presenting cells, complement cascades, and the induction of chemokine secretion. Despite its well-known toxicity, particularly for the central nervous system, Al has been included in many vaccine preparations for many years because it has been calculated that the total amount of Al that children receive when they are given all the vaccine doses included in the national immunization schedules is significantly lower than that associated with neurotoxicity (94). Impaired speech, apraxia, concentration problems, dementia, depression, and fatigue were described in adults who had been exposed for long time to Al because of foundry work or those who were chronically dialyzed with solution containing relevant amounts of Al (95, 96). In pediatric patients, developmental delay has been described in preterm infants maintained for long periods on parenteral nutrition with solutions with high Al content (97). On the other hand, attempts to correlate the development of autism spectrum disorders or Alzheimer's disease with Al exposure have failed because some studies did not reveal any true association (98, 99), and positive studies in both animals and children (100–102) had several methodological limitations that made it impossible to draw reliable conclusions (103).

Despite a lack of data supporting damage from Al in subjects receiving vaccines containing this adjuvant, the question of whether vaccines containing Al can be dangerous continues to be raised (104). One of the most frequently reported reasons for the elimination of Al from vaccines is the risk that Al could induce the hyperactivation of the immune system, leading to an autoimmune disease. Autoimmune/autoinflammatory syndrome induced by adjuvants (ASIA) would be the best demonstration of the potentially dangerous role of Al in this regard. With the definition of ASIA, several different clinical entities that are unified by the previous administration of an adjuvant are included. Sick building syndrome, silicosis, Gulf war syndrome and macrophagic myofasciitis (MMF) have been described (105, 106). Moreover, it was suggested that lymphoma, Sjogren syndrome, narcolepsy, and phospholipid syndrome can be included in the ASIA group (107–109). Arthralgia, myalgia, and chronic fatigue were the most frequently reported symptoms in most of these conditions (110). However, the real association between Al and ASIA development has not been definitively ascertained. Most of the studies that seem to indicate a relationship between adjuvants and ASIA are burdened by strong methodological limitations. Criteria for the diagnosis of ASIA are extremely coarse and include signs and symptoms that can occur in several non-immunologically based diseases (111, 112). Moreover, some genetic characteristics that are considered specific to ASIA patients can be commonly found in individuals with other autoimmune diseases. Consequently, it seems likely that in many epidemiological evaluations, most of the cases considered ASIA had a different disease, totally independent from autoimmunity, making the results totally unreliable.

However, several other factors make the correlation between Al and ASIA highly unlikely. Association between vaccines with Al and autoimmune diseases is extremely uncommon even when a temporal association between vaccine administration and disease development seems to suggest a linkage (113). The administration of vaccines containing Al does not worsen the clinical course of autoimmune diseases, contrary to what would be expected if Al could evoke autoimmunity (114). Intradermal administration of antigen preparations containing Al for the treatment of allergic diseases is not associated with any autoimmune manifestation despite the long-term exposure to a non-marginal amount of the metal and the administration in a site favoring a strong immune response (115). Finally, some data collected in children with MMF and associated brain damage seem to suggest that Al distribution into the brain and related CNS alterations are strongly influenced by some genetic characteristics (patients in the HLA-DRB1^*^01 group or with increased expression of the monocyte chemoattractant protein-1 gene) (116, 117). This indicates the importance of genetics rather than autoimmunity in conditioning Al-related CNS disease development. On the other hand, MMF can also be diagnosed in patients who did not receive an adjuvanted vaccine, which suggests that causes other than vaccines can be associated with ASIA (118).

## Mercury-Related Neuropathies

It has been evidenced in both experimental animals and humans that exposure to high mercury levels could be neurotoxic. For many years, mercury has been included in vaccines in the form of thimerosal (ethylmercury bound to thiosalicylate) because of its preservative action. At the end of last century, it was calculated that infants given all the vaccines recommended in the first 6 months of life received an amount of mercury that exceeded the maximum acceptable daily intake according to the Environmental Protection Agency for methylmercury. Although no well-conducted study had shown that fully immunized children were at increased risk of developing any type of neurological disease, including autism and delayed development, this led several scientific societies to recommend that thimerosal was eliminated from all the vaccines (119). This decision was debated by some experts who highlighted that in vaccines, mercury was present as ethylmercury and not methylmercury. As ethylmercury had a significantly more rapid metabolism and excretion than methylmercury (120), it was concluded that the exposure to mercury of immunized children was significantly lower than that calculated and the removal of thimerosal was not truly needed. Despite this, thimerosal was eliminated from all the vaccines and presently remains only in preparations used in some developing countries and in multidose vial influenza vaccines.

However, to evaluate whether mercury contained in vaccines could cause problems, several studies were performed. Regarding those specifically planned to demonstrate autoimmunity development, it was shown that subjects exposed to mercury through foods or work could have cellular and humoral signs of immune system activation and, in some cases, high levels of serum autoantibodies or anti-nuclear antibodies (121). This was considered evidence of a potential role of mercury in the determination of autoimmunity (122). Moreover, some mercury-related modifications were strictly associated with well-defined genetic characteristics, quite like those found in subjects with autoimmune diseases (123, 124). Despite these findings, no data capable of showing a causative role for mercury in autoimmune diseases are presently available. On the other hand, all the well-conducted epidemiological studies carried out to show whether thimerosal could cause neurological problems did not show any association between this preservative and autism or development delay (125–127). In one case, it has even been highlighted that exposure to mercury can represent an event conducive to better neurological development (127). In particular, it has been highlighted that exposure to mercury during intrauterine life can lead to better language development skills even if it can lead to less attention and less valid executive functions. Exposure to mercury in the period from birth to the end of the 7th month seems to ensure better motor coordination, higher attention and more efficient executive skills. The lack of negative elements and even the possibility of positive data explain why major international scientific institutions such as the WHO, FDA and EMA concluded that a correlation between thimerosal content in vaccines and the development of neurological diseases could be excluded (128–130).

## Conclusions

Most of the associations between vaccines and nervous system autoimmune syndromes that have been reported as severe adverse events following immunization are no longer evidenced when well-conducted epidemiological studies are carried out. Biological plausibility of the autoimmune mechanisms associated with the temporal proximity between vaccine administration and disease development can be misleading. Moreover, bad epidemiological evaluations can worsen the final judgement and lead to conclusions very far from reality. Although the rarity of autoimmune diseases makes it difficult to strictly exclude that, albeit exceptionally, some vaccines may induce an autoimmune neurological disease, no definitive demonstration of a potential role of vaccines in causing autoimmune neurological syndromes is presently available. The fear of neurological autoimmune disease cannot limit the use of the most important preventive measure presently available against infectious diseases.

## Author Contributions

NP and SE co-wrote the manuscript and critically revised the text with substantial scientific contributions. All authors approved the final version of the manuscript.

## Conflict of Interest

The authors declare that the research was conducted in the absence of any commercial or financial relationships that could be construed as a potential conflict of interest.

## References

[1] Doherty M Buchy P Standaert B Giaquinto C Prado-Cohrs D. Vaccine impact: benefits for human health. Vaccine. (2016) 34:6707–14. 10.1016/j.vaccine.2016.10.025 27773475

[2] Plotkin SA Plotkin SL A short history of vaccination. In: Plotkin SA Orenstein WA Offit PA, editors. Vaccines. 6th ed. Philadelphia, PA: WB Saunders (2012). p. 1−13. 10.1016/B978-1-4557-0090-5.00017-3

[3] Hajj Hussein I Chams N Chams S El Sayegh S Badran R Raad M. Vaccines through centuries: major cornerstones of global health. Front Public Health. (2015) 3:269. 10.3389/fpubh.2015.00269 26636066 PMC4659912

[4] Dubé E Vivion M MacDonald NE. Vaccine hesitancy, vaccine refusal and the anti-vaccine movement: influence, impact and implications. Expert Rev Vaccines. (2015) 14:99–117. 10.1586/14760584.2015.964212 25373435

[5] World Health Organization Emergencies Preparedness, Response. Measles-Global Situation. Available online at: https://www.who.int/csr/don/26-november-2019-measles-global_situation/en/ (accessed March 3, 2020).

[6] Kennedy J. Vaccine hesitancy: a growing concern. Paediatr Drugs. (2020) 22:105–11. 10.1007/s40272-020-00385-4 32072472

[7] Principi N Esposito S. Adverse events following immunization: real causality and myths. Expert Opin Drug Saf. (2016) 15:825–35. 10.1517/14740338.2016.1167869 26986067

[8] Nguyen XH Saoudi A Liblau RS. Vaccine-associated inflammatory diseases of the central nervous system: from signals to causation. Curr Opin Neurol. (2016). 29:362–71. 10.1097/WCO.0000000000000318 27023738

[9] Committee to Review Adverse Effects of Vaccines Institute of Medicine. In: Stratton K Ford A Rusch E Clayton EW, editors. Adverse Effects of Vaccines: Evidence and Causality. Washington, DC: National Academies Press (2011). 24624471

[10] Galetta KM Bhattacharyya S. Multiple sclerosis and autoimmune neurology of the central nervous system. Med Clin North Am. (2019) 103:325–36. 10.1016/j.mcna.2018.10.004 30704684

[11] Hu W Lucchinetti CF. The pathological spectrum of CNS inflammatory demyelinating diseases. Semin Immunopathol. (2009) 31:439–53. 10.1007/s00281-009-0178-z 19779719

[12] Institute of Medicine (US) Forum on Microbial Threats. Knobler SL O'Connor S Lemon SM editors. The Infectious Etiology of Chronic Diseases: Defining the Relationship, Enhancing the Research, and Mitigating the Effects: Workshop Summary. Washington, DC: National Academies Press (2004). 22379643

[13] Kerr DA Ayetey H. Immunopathogenesis of acute transverse myelitis. Curr Opin Neurol. (2002) 15:339–47. 10.1097/00019052-200206000-00019 12045735

[14] Yadav SK Mindur JE Ito K Dhib-Jalbut S. Advances in the immunopathogenesis of multiple sclerosis. Curr Opin Neurol. (2015) 28:206–19. 10.1097/WCO.0000000000000205 25887768

[15] Jakimovski D Weinstock-Guttman B Ramanathan M Dwyer MG Zivadinov R. Infections, vaccines and autoimmunity: a multiple sclerosis perspective. Vaccines (Basel). (2020) 8:E50. 10.3390/vaccines8010050 32012815 PMC7157658

[16] Marshall E. A shadow falls on hepatitis B vaccination effort. Science. (1998) 281:630–1. 10.1126/science.281.5377.630 9714670

[17] Ascherio A Zhang SM Hernán MA Olek MJ Coplan PM Brodovicz K. Hepatitis B vaccination and the risk of multiple sclerosis. N Engl J Med. (2001) 344:327–32. 10.1056/NEJM200102013440502 11172163

[18] DeStefano F Verstraeten T Chen RT. Hepatitis B vaccine and risk of multiple sclerosis. Expert Rev Vaccines. (2002) 1:461–6. 10.1586/14760584.1.4.461 12901584

[19] Sadovnick AD Scheifele DW. School-based hepatitis B vaccination programme and adolescent multiple sclerosis. Lancet. (2000) 355:549–50. 10.1016/S0140-6736(99)02991-8 10683009

[20] Mikaeloff Y Caridade G Suissa S Tardieu M. Hepatitis B vaccine and the risk of CNS inflammatory demyelination in childhood. Neurology. (2009) 72:873–80. 10.1212/01.wnl.0000335762.42177.07 18843097

[21] Mouchet J Bégaud B. Hepatitis B vaccination and central demyelination - history, description and observed/expected analyses of 624 cases reported to the French pharmacovigilance over a 20-year period. Vaccine. (2019) 37:2142–8. 10.1016/j.vaccine.2019.02.046 30851966

[22] Le Houézec D. Evolution of multiple sclerosis in France since the beginning of hepatitis B vaccination. Immunol Res. (2014) 60:219–25. 10.1007/s12026-014-8574-4 25395338 PMC4266455

[23] Meggiolaro A Migliara G La Torre G. Association between Human Papilloma Virus (HPV) vaccination and risk of multiple sclerosis: a systematic review. Hum Vaccin Immunother. (2018) 14:1266–74. 10.1080/21645515.2017.1423155 29333935 PMC5989905

[24] Mouchet J Salvo F Raschi E Poluzzi E Antonazzo IC De Ponti F. Human papillomavirus vaccine and demyelinating diseases-A systematic review and meta-analysis. Pharmacol Res. (2018) 132:108–18. 10.1016/j.phrs.2018.04.007 29665426

[25] Willame C Gadroen K Bramer W Weibel D Sturkenboom M. Systematic review and meta-analysis of postlicensure observational studies on human papillomavirus vaccination and autoimmune and other rare adverse events. Pediatr Infect Dis J. (2020) 39:287–93 10.1097/INF.0000000000002569 31876615

[26] Bardage C Persson I Ortqvist A Bergman U Ludvigsson JF Granath F. Neurological and autoimmune disorders after vaccination against pandemic influenza A (H1N1) with a monovalent adjuvanted vaccine: population based cohort study in Stockholm, Sweden. BMJ. (2011) 343:d5956. 10.1136/bmj.d5956 21994316 PMC3192001

[27] Kurland LT Molgaard CA Kurland EM Wiederholt WC Kirkpatrick JW. Swine flu vaccine and multiple sclerosis. JAMA. (1984) 251:2672–5. 10.1001/jama.251.20.2672 6325730

[28] Baxter R Lewis E Goddard K Fireman B Bakshi N De Stefano F. Acute demyelinating events following vaccines: a case-centered analysis. Clin Infect Dis. (2016) 63:1456–62. 10.1093/cid/ciw607 27585798 PMC6708556

[29] Mailand MT Frederiksen JL. Vaccines and multiple sclerosis: a systematic review. J Neurol. (2017) 264:1035–50. 10.1007/s00415-016-8263-4 27604618

[30] Langer-Gould A Qian L Tartof SY Brara SM Jacobsen SJ Beaber BE. Vaccines and the risk of multiple sclerosis and other central nervous system demyelinating diseases. JAMA Neurol. (2014) 71:1506–13. 10.1001/jamaneurol.2014.2633 25329096

[31] Chen Y Ma F Xu Y Chu X Zhang J. Vaccines and the risk of acute disseminated encephalomyelitis. Vaccine. (2018) 36:3733–9. 10.1016/j.vaccine.2018.05.063 29784468

[32] Sejvar JJ Baughman AL Wise M Morgan OW. Population incidence of Guillain-Barré syndrome: a systematic review and meta-analysis. Neuroepidemiology. (2011) 36:123–33. 10.1159/000324710 21422765 PMC5703046

[33] Poropatich KO Walker CL Black RE. Quantifying the association between campylobacter infection and guillain-Barré syndrome: a systematic review. J Health Popul Nutr. (2010) 28:545–52. 10.3329/jhpn.v28i6.6602 21261199 PMC2995022

[34] Hadden RDM Karch H Hartung HP Zielasek J Weissbrich B Schubert J. Preceding infections, immune factors, and outcome in Guillain-Barré syndrome. Neurology. (2001) 56:758–65. 10.1212/WNL.56.6.758 11274311

[35] Jacobs BC Rothbarth PH van der Meché FGA Herbrink P Schmitz PI de Klerk MA. The spectrum of antecedent infections in Guillain-Barré syndrome: a case-control study. Neurology. (1998) 51:1110–5. 10.1212/WNL.51.4.1110 9781538

[36] Yuki N Hartung HP. Guillain-Barré syndrome. N Engl J Med. (2012) 366:2294–304. 10.1056/NEJMra1114525 22694000

[37] Cao-Lormeau VM Blake A Mons S Lastère S Roche C Vanhomwegen J. Guillain-Barré syndrome outbreak associated with Zika virus infection in French Polynesia: a case-control study. Lancet. (2016) 387:1531–9. 10.1016/S0140-6736(16)00562-6 26948433 PMC5444521

[38] Tatarelli P Garnero M Del Bono V Camera M Schenone A Grandis M. Guillain-Barré syndrome following chickenpox: a case series. Int J Neurosci. (2016) 126:478–9. 10.3109/00207454.2015.1033621 26000930

[39] Filia A Lauria G. Guillain-Barré syndrome following measles infection: case report and review of the literature. Neurol Sci. (2014) 35:2017–8. 10.1007/s10072-014-1841-2 24870223

[40] Mori M Kuwabara S Miyake M Noda M Kuroki H Kanno H. Haemophilus influenzae infection and Guillain-Barré syndrome. Brain. (2000) 123:2171–8. 10.1093/brain/123.10.2171 11004133

[41] Wakerley BR Uncini A Yuki N. GBS Classification Group. Guillain-Barré and miller fisher syndromes–new diagnostic classification. Nat Rev Neurol. (2014) 10:537–44. 10.1038/nrneurol.2014.138 25072194

[42] Allen D Giannopoulos K Gray I Gregson N Makowska A Pritchard J. Antibodies to peripheral nerve myelin proteins in chronic inflammatory demyelinating polyradiculoneuropathy. J Peripher Nerv Syst. (2005) 10:174–80. 10.1111/j.1085-9489.2005.0010207.x 15958128

[43] Ziganshin RH Ivanova OM Lomakin YA Belogurov AA Jr Kovalchuk SI Azarkin IV. The pathogenesis of the Demyelinating Form of Guillain-Barre Syndrome (GBS): proteo-peptidomic and immunological profiling of physiological fluids. Mol Cell Proteomics. (2016) 15:2366–78. 10.1074/mcp.M115.056036 27143409 PMC4937510

[44] Irie S Saito T Nakamura K Kanazawa N Ogino M Nukazawa T. Association of anti-GM2 antibodies in Guillain-Barré syndrome with acute cytomegalovirus infection. J Neuroimmunol. (1996) 68:19–26. 10.1016/0165-5728(96)00059-8 8784256

[45] Kusunoki S Chiba A Hitoshi S Takizawa H Kanazawa I. Anti-Gal-C antibody in autoimmune neuropathies subsequent to mycoplasma infection. Muscle Nerve. (1995) 18:409–13. 10.1002/mus.880180407 7715626

[46] Yuki N Yamada M Koga M Odaka M Susuki K Tagawa Y. Animal model of axonal Guillain-Barré syndrome induced by sensitization with GM1 ganglioside. Ann Neurol. (2001) 49:712–20. 10.1002/ana.1012 11409422

[47] Moyano AL Comín R Lardone RD Alaniz ME Theaux R Irazoqui FJ. Validation of a rabbit model of neuropathy induced by immunization with gangliosides. J Neurol Sci. (2008) 272:110–4. 10.1016/j.jns.2008.05.006 18573503

[48] Caporale CM Capasso M Luciani M Prencipe V Creati B Gandolfi P. Experimental axonopathy induced by immunization with *Campylobacter jejuni* lipopolysaccharide from a patient with Guillain-Barré syndrome. J Neuroimmunol. (2006) 174:51. 10.1016/j.jneuroim.2005.12.005 16516981

[49] Principi N Esposito S. Vaccine-preventable diseases, vaccines and Guillain-Barre' syndrome. Vaccine. (2019) 37:5544–50. 10.1016/j.vaccine.2018.05.119 29880241

[50] DeStefano F Bodenstab HM Offit PA. Principal controversies in vaccine safety in the United States. Clin Infect Dis. (2019) 69:726–31. 10.1093/cid/ciz135 30753348

[51] Merck Sharp & Dohme Limited. MMRVAXPRO. Available online at: https://www.medicines.org.uk/emc/product/6307 (accessed March 3, 2020).

[52] Morris K Rylance G. Guillain-Barré syndrome after measles, mumps, and rubella vaccine. Lancet. (1994) 343:60. 10.1016/S0140-6736(94)90917-2 7905077

[53] Jonse R II Romanus V Böttiger M Sandzelius G Polyradikulit I. An slutningen till vaccination mot morbilli, parotit och rubella. Läkartidningen. (1984) 81:1636–7. 6717184

[54] Böttiger M Christenson B Romanus V Taranger J Strandell A. Swedish experience of two dose vaccination programme aiming at eliminating measles, mumps, and rubella. BMJ. (1987) 295:1264–7. 10.1136/bmj.295.6608.1264 3120971 PMC1248321

[55] Fescharek R Quast U Maass G Merkle W Schwarz S. Measles mumps vaccination in the FRG: an empirical analysis after 14 years of use. II. tolerability and analysis of spontaneously reported side effects. Vaccine. (1990) 8:446–56. 10.1016/0264-410X(90)90245-H 2251871

[56] Plesner AM. Gait disturbances after measles, mumps, and rubella vaccine. Lancet. (1995) 345:316. 10.1016/S0140-6736(95)90302-X 7837872

[57] Institute of Medicine Adverse Events Associated With Childhood Vaccines: Evidence Bearing on Causality. Measles and Mumps Vaccines. Washington, DC: National Academy Press (2011).

[58] Patja A Paunio M Kinnunen E Junttila O Hovi T Peltola H. Risk of Guillain-Barré syndrome after measles-mumps-rubella vaccination. J Pediatr. (2001) 138:250–4. 10.1067/mpd.2001.111165 11174624

[59] Esteghamati A Gouya MM Keshtkar AA Mahoney F. Relationship between occurrence of Guillain-Barre syndrome and mass campaign of measles and rubella immunization in Iranian 5–14 years old children. Vaccine. (2008) 26:5058–61. 10.1016/j.vaccine.2008.07.014 18662736

[60] da Silveira CM Salisbury DM de Quadros CA. Measles vaccination and Guillain-Barré syndrome. Lancet. (1997) 349:14–6. 10.1016/S0140-6736(96)07408-9 8988116

[61] Miranda S Chaignot C Collin C Dray-Spira R Weill A Zureik M. Human papillomavirus vaccination and risk of autoimmune diseases: a large cohort study of over 2million young girls in France. Vaccine. (2017) 35:4761–8. 10.1016/j.vaccine.2017.06.030 28750853

[62] World Health Organization Safety of HPV Vaccines. Available online at: http://www.who.int/vaccine_safety/committee/topics/hpv/Dec_2015/en/ (accessed February 28, 2020).

[63] Grimaldi-Bensouda L Rossignol M Koné-Paut I Krivitzky A Lebrun-Frenay C Clet J. Risk of autoimmune diseases and human papilloma virus (HPV) vaccines: six years of case-referent surveillance. J Autoimmun. (2017) 79:84–90. 10.1016/j.jaut.2017.01.005 28190705

[64] Andrews N Stowe J Miller E. No increased risk of Guillain-Barré syndrome after human papilloma virus vaccine: a self-controlled case-series study in England. Vaccine. (2017) 35:1729–32. 10.1016/j.vaccine.2017.01.076 28245941

[65] Angelo MG David MP Zima J Baril L Dubin G Arellano F. Pooled analysis of large and long-term safety data from the human papillomavirus-16/18-AS04-adjuvanted vaccine clinical trial programme. Pharmacoepidemiol Drug Saf. (2014) 23:466–79. 10.1002/pds.3554 24644063 PMC4230467

[66] Gee J Naleway A Shui I Baggs J Yin R Li R. Monitoring the safety of quadrivalent human papillomavirus vaccine: findings from the vaccine safety datalink. Vaccine. (2011) 29:8279–84. 10.1016/j.vaccine.2011.08.106 21907257

[67] Markowitz LE Dunne EF Saraiya M Chesson HW Curtis CR Gee J Human papillomavirus vaccination: recommendations of the Advisory Committee on Immunization Practices (ACIP). MMWR Recomm Rep. (2014) 63:1–30. 25167164

[68] World Health Organization. Meeting of the global advisory committee on vaccine safety, 7–8 June 2017. Weekly Epidemiol Record. (2017) 92:393–404. 28707463

[69] Centers for Disease Control and Prevention. Guillain-Barré syndrome among recipients of Menactra meningococcal conjugate vaccine–United States, June-July 2005. MMWR Morb Mortal Wkly Rep. (2005). 54:1023–5. 16224452

[70] Cho B Clark TA Messonnier NE. MCV vaccination in the presence of vaccine associated Guillain-Barré Syndrome risk: a decision analysis approach. Vaccine. (2010) 28:817–22. 10.1016/j.vaccine.2009.10.050 19879992

[71] Centers for Disease Control and Prevention. Update: Guillain-Barré syndrome among recipients of Menactra meningococcal conjugate vaccine—United States, October 2005– February 2006. MMWR Morb Mortal Wkly Rep. (2006) 55:364–6. 16601664

[72] Hansen J Zhang L Klein NP Robertson CA Decker MD Greenberg DP. Post-licensure safety surveillance study of routine use of quadrivalent meningococcal diphtheria toxoid conjugate vaccine. Vaccine. (2017) 35:6879–84. 10.1016/j.vaccine.2017.09.032 28941623

[73] Velentgas P Amato AA Bohn RL Chan KA Cochrane T Funch DP. Risk of Guillain-Barré syndrome after meningococcal conjugate vaccination. Pharmacoepidemiol Drug Saf. (2012) 21:1350–8. 10.1002/pds.3321 22807266

[74] Yih WK Weintraub E Kulldorff M. No risk of Guillain-Barré syndrome found after meningococcal conjugate vaccination in two large cohort studies. Pharmacoepidemiol Drug Saf. (2012) 21:1359–60. 10.1002/pds.3353 23225672

[75] Cohn AC MacNeil JR Clark TA Ortega-Sanchez IR Briere EZ Meissner HC. Prevention and control of meningococcal disease: recommendations of the Advisory Committee on Immunization Practices (ACIP). MMWR Recomm Rep. (2013) 62:1–28. 23515099

[76] Langmuir AD Bregman DJ Kurland LT Nathanson N Victor M. An epidemiologic and clinical-evaluation of Guillain-Barre-syndrome reported in association with the administration of swine influenza vaccines. Am J Epidemiol. (1984) 119:841–79. 10.1093/oxfordjournals.aje.a113809 6328974

[77] Institute of Medicine Immunization Safety Review: Influenza Vaccines and Neurological Complications. Washington, DC: The National Academies Press (2003). 25057683

[78] Baxter R Bakshi N Fireman B Lewis E Ray P Vellozzi C. Lack of association of Guillain-Barre syndrome with vaccinations. Clin Infect Dis. (2013) 57:197–204. 10.1093/cid/cit222 23580737

[79] Burwen DR Ball R Bryan WW Izurieta HS La Voie L Gibbs NA. Evaluation of Guillain-Barre syndrome among recipients of influenza vaccine in 2000 and 2001. Am J Prev Med. (2010) 39:296–304. 10.1016/j.amepre.2010.05.022 20837279

[80] Greene SK Kulldorff M Lewis EM Li R Yin R Weintraub ES. Near realtime surveillance for influenza vaccine safety: proof-of-concept in the vaccine safety datalink project. Am J Epidemiol. (2010) 171:177–88. 10.1093/aje/kwp345 19965887 PMC2878099

[81] Hughes RAC Cornblath DR Guillain-Barre syndrome—reply. Lancet. (2006) 367:473–4. 10.1016/S0140-6736(06)68176-2

[82] Juurlink DN Stukel TA Kwong J Kopp A McGeer A Upshur RE. Guillain-Barre syndrome after influenza vaccination in adults—a population-based study. Arch Int Med. (2006) 166:2217–21. 10.1001/archinte.166.20.2217 17101939

[83] Kaplan JE Katona P Hurwitz ES Schonberger LB. Guillain-Barrè syndrome in the United States, 1979–1980 and 1980–1981—lack of an association with influenza vaccination. JAMA. (1982) 248:698–700. 10.1001/jama.248.6.698 7097920

[84] Lasky T Terracciano GJ Magder L Koski CL Ballesteros M Nash D. The Guillain-Barre syndrome and the 1993 and 1993–1994 influenza vaccines. N Engl J Med. (1998) 339:1797–802. 10.1056/NEJM199812173392501 9854114

[85] Salmon DA Proschan M Forshee R Gargiullo P Bleser W Burwen DR. Association between Guillain-Barré syndrome and influenza A (H1N1) 2009 monovalent inactivated vaccines in the USA: a meta-analysis. Lancet. (2013) 381:1461–8. 10.1016/S0140-6736(12)62189-8 23498095

[86] De Wals P Deceuninck G Toth E Boulianne N Brunet D Boucher RM. Risk of Guillain-Barré syndrome following H1N1 influenza vaccination in Quebec. JAMA. (2012) 308:175–81. 10.1001/jama.2012.7342 22782419

[87] Dieleman J Romio S Johansen K Weibel D Bonhoeffer J Sturkenboom M. Guillain-Barre syndrome and adjuvanted pandemic influenza A (H1N1) 2009 vaccine: multinational case-control study in Europe. BMJ. (2011) 343:d3908. 10.1136/bmj.d3908 21750072 PMC3134565

[88] Sandhu SK Hua W MaCurdy TE Franks RL Avagyan A Kelman J. Near realtime surveillance for Guillain-Barré syndromeafter influenza vaccination among the medicare population, 2010/11 to 2013/14. Vaccine. (2017) 35:2986–92. 10.1016/j.vaccine.2017.03.087 28449973

[89] Schonberger LB Bregman DJ Sullivan-Bolyai JZ Keenlyside RA Ziegler DW Retailliau HF. Guillain-Barre syndrome following vaccination in the national influenza immunization program, United States, 1976–1977. Am J Epidemiol. (1979) 110:105–23. 10.1093/oxfordjournals.aje.a112795 463869

[90] Vellozzi C Iqbal S Stewart B Tokars J DeStefano F. Cumulative risk of Guillain-Barré syndrome among vaccinated and unvaccinated populations during the 2009. H1N1 influenza pandemic. Am J Public Health. (2014) 104:696–701. 10.2105/AJPH.2013.301651 24524517 PMC4025712

[91] Dodd CN Romio SA Black S Vellozzi C Andrews N Sturkenboom M. International collaboration to assess the risk of Guillain Barré Syndrome following Influenza A (H1N1) 2009 monovalent vaccines. Vaccine. (2013) 31:4448–58. 10.1016/j.vaccine.2013.06.032 23770307

[92] Kwong JC Vasa PP Campitelli MA Hawken S Wilson K Rosella LC. Risk of Guillain-Barré syndrome after seasonal influenza vaccination and influenza health-care encounters: a self-controlled study. Lancet Infect Dis. (2013) 13:769–76. 10.1016/S1473-3099(13)70104-X 23810252

[93] Eickhoff TC Myers M. Workshop summary Aluminum in vaccines. Vaccine. (2002) 20(Suppl. 3):S1–4. 10.1016/S0264-410X(02)00163-9 12184358

[94] Principi N Esposito S. Aluminum in vaccines: does it create a safety problem? Vaccine. (2018) 36:5825–31. 10.1016/j.vaccine.2018.08.036 30139653

[95] Kiesswetter E Schaeper M Buchta M Schaller KH Rossbach B Scherhag H. Longitudinal study on potential neurotoxic effects of aluminum: I. assessment of exposure and neurobehavioral performance of Al welders in the train and truck construction industry over 4 years. Int Arch Occup Environ Health. (2007) 81:41–67. 10.1007/s00420-007-0191-2 17522885

[96] Kiesswetter E Schäper M Buchta M Schaller KH Rossbach B Kraus T. Longitudinal study on potential neurotoxic effects of aluminum: II. assessment of exposure and neurobehavioral performance of Al welders in the automobile industry over 4 years. Int Arch Occup Environ Health. (2009) 82:1191–210. 10.1007/s00420-009-0414-9 19326140

[97] Fewtrell MS Edmonds CJ Isaacs E Bishop NJ Lucas A. Aluminum exposure from parenteral nutrition in preterm infants and later health outcomes during childhood and adolescence. Proc Nutr Soc. (2011) 70:299–304. 10.1017/S0029665111000498 21781356

[98] Virk SA Eslick GD. Occupational exposure to aluminum and Alzheimer disease: a meta-analysis. J Occup Environ Med. (2015) 57:893–6. 10.1097/JOM.0000000000000487 26247643

[99] Salib E Hillier V. A case-control study of Alzheimer's disease and aluminum occupation. Br J Psychiatry. (1996) 168:244–9. 10.1192/bjp.168.2.244 8837919

[100] Shaw CA Li Y. Tomljenovic L Administration of aluminum to neonatal mice in vaccine-relevant amounts is associated with adverse long-term neurological outcomes. J Inorg Biochem. (2013) 128:237–44. 10.1016/j.jinorgbio.2013.07.022 23932735

[101] Tomljenovic L Shaw CA. Do aluminum vaccine adjuvants contribute to the rising prevalence of autism? J Inorg Biochem. (2011) 105:1489–99. 10.1016/j.jinorgbio.2011.08.008 22099159

[102] Tomljenovic L Shaw CA. Aluminum vaccine adjuvants: are they safe? Cur Med Chem. (2011) 18:2630–7. 10.2174/092986711795933740 21568886

[103] Karwowski MP Stamoulis C Wenren LM Faboyede GM Quinn N Gura KM. Blood and hair aluminum levels, vaccine history, and early infant development: a cross-sectional study. Acad Pediatr. (2018) 18:161–5. 10.1016/j.acap.2017.09.003 28919482

[104] Miller NZ Aluminum Miller's review of critical vaccine studies. 400 important scientific papers summarized for parents and researchers. Santa Fe, NM: New Atlantean Press (2016). p. 44–63.

[105] Shoenfeld Y Agmon-Levin N. ‘ASIA’—autoimmune/inflammatory syndrome induced by adjuvants. J Autoimmun. (2011) 36:4–8. 10.1016/j.jaut.2010.07.003 20708902

[106] Watad A Quaresma M Brown S Cohen Tervaert JW Rodriguez-Pint I Cervera R. Autoimmune/inflammatory syndrome induced by adjuvants (Shoenfeld's syndrome)—an update. Lupus. (2017) 26:675–81. 10.1177/0961203316686406 28059022

[107] Colafrancesco S Perricone C Priori R Valesini G Shoenfeld Y. Sjogren's syndrome: another facet of the autoimmune/inflammatory syndrome induced by adjuvants (ASIA). J Autoimmun. (2014) 51:10–6. 10.1016/j.jaut.2014.03.003 24774584

[108] Arango MT Kivity S Shoenfeld Y. Is narcolepsy a classical autoimmune disease? Pharmacol Res. (2015) 92:6–12. 10.1016/j.phrs.2014.10.005 25447795

[109] Butnaru D Shoenfeld Y. Adjuvants and lymphoma risk as part of the ASIA spectrum. Immunol Res. (2015) 61:79–89. 10.1007/s12026-014-8622-0 25582758

[110] Watad A Quaresma M Bragazzi NL Cervera R Tervaert JWC Amital H. The autoimmune/inflammatory syndrome induced by adjuvants (ASIA)/Shoenfeld's syndrome: descriptive analysis of 300 patients from the international ASIA syndrome registry. Clin Rheumatol. (2018) 37:483–93. 10.1007/s10067-017-3748-9 28741088

[111] Esposito S Prada E Mastrolia MV Tarantino G Codecà C Rigante D. Autoimmune/inflammatory syndrome induced by adjuvants (ASIA): clues and pitfalls in the pediatric background. Immunol Res. (2014) 60:366–75. 10.1007/s12026-014-8586-25395340

[112] Ameratunga R Gillis D Gold M Linneberg A Elwood JM. Evidence refuting the existence of autoimmune/autoinflammatory syndrome induced by adjuvants (ASIA). J Allergy Clin Immunol Pract. (2017) 5:1551–5. 10.1016/j.jaip.2017.06.033 28888842

[113] Wraith DC Goldman M Lambert PH. Vaccination and autoimmune disease: what is the evidence? Lancet. (2003) 362:1659–66. 10.1016/S0140-6736(03)14802-7 14630450

[114] Kuruma KA Borba EF Lopes MH de Carvalho JF Bonfa E. Safety and efficacy of hepatitis B vaccine in systemic lupus erythematosus. Lupus. (2007) 16:350–4. 10.1177/0961203307078225 17576737

[115] Ameratunga R Già Citato Linneberg A Jacobsen RK Jespersen L Abildstrom SZ. Association of subcutaneous allergen-specific immunotherapy with incidence of autoimmune disease, ischemic heart disease, and mortality. J Allergy Clin Immunol. (2012) 129:413–9. 10.1016/j.jaci.2011.09.007 22004944

[116] Guis S Pellissier JF Nicoli F Reviron D Mattei JP Gherardi RK. HLADRB1^*^01 and macrophagic myofasciitis. Arthritis Rheum. (2002) 46:2535–7. 10.1002/art.10465 12355502

[117] Deshmane SL Kremlev S Amini S Sawaya BE. Monocyte chemoattractant protein-1 (MCP-1): an overview. J Interferon Cytokine Res. (2009) 29:313–26. 10.1089/jir.2008.0027 19441883 PMC2755091

[118] Park JH Na KS Park YW Paik SS Yoo DH. Macrophagic myofasciitis unrelated to vaccination. Scand J Rheumatol. (2005) 34:65–7. 10.1080/0300974051007913 15903029

[119] US Food and Drug Administration Thimerosal and Vaccines. Available online at: https://www.fda.gov/vaccines-blood-biologics/safety-availability-biologics/thimerosal-and-vaccines (accessed March 3, 2020).

[120] Dórea JG Farina M Rocha JB. Toxicity of ethylmercury (and Thimerosal): a comparison with methylmercury. J Appl Toxicol. (2013) 33:700–11. 10.1002/jat.2855 23401210

[121] Pollard KM Cauvi DM Toomey CB Hultman P Kono DH. Mercury-induced inflammation and autoimmunity. Biochim Biophys Acta Gen Subj. (2019) 1863:129299. 10.1016/j.bbagen.2019.02.001 30742953 PMC6689266

[122] Bjørklund G Peana M Dadar M Chirumbolo S Aaseth J Martins N. Mercury-induced autoimmunity: drifting from micro to macro concerns on autoimmune disorders. Clin Immunol. (2020) 213:108352. 10.1016/j.clim.2020.108352 32032765

[123] Hu H Moller G Abedi-Valugerdi M. Major histocompatibility complex class II antigens are required for both cytokine production proliferation induced by mercuric chloride *in vitro*. J Autoimmun. (1997) 10:441–6. 10.1006/jaut.1997.9997 9376071

[124] Hanley GA Schiffenbauer J Sobel ES. Class II haplotype differentially regulates immune response in HgCl2- treated mice. Clin Immunol Immunopathol. (1997) 84:328–37. 10.1006/clin.1997.4405 9281392

[125] Heron J Golding J. Thimerosal exposure in infants and developmental disorders: a prospective cohort study in the United Kingdom does not support a causal association. Pediatrics. (2004) 114:577–83. 10.1542/peds.2003-1176-L 15342824

[126] Price CS Thompson WW Goodson B Weintraub ES Croen LA Hinrichsen VL. Prenatal and infant exposure to thimerosal from vaccines and immunoglobulins and risk of autism. Pediatrics. (2010) 126:656–64. 10.1542/peds.2010-0309 20837594

[127] Thompson WW Price C Goodson B Shay DK Benson P Hinrichsen VL. Early thimerosal exposure and neuropsychological outcomes at 7 to 10 years. N Engl J Med. (2007) 357:1281–92. 10.1056/NEJMoa071434 17898097

[128] World Health Organization Global vaccine safety. Thiomersal and vaccines: questions and answers. Available online at: http://www.who.int/vaccine_safety/committee/topics/thiomersal/questions/en/. (accessed March 3, 2020).

[129] Food and Drug Administration Thimerosal in vaccines. Available online at: http://www.fda.gov/BiologicsBloodVaccines/SafetyAvailability/VaccineSafety/UCM096228#saf (accessed March 3, 2020).

[130] The European Agency for the Evaluation of Medicinal Products EMEA public statement on thiomersal in vaccines for human use. Recent evidences support safety of thiomersal-containing vaccines. Available online at: http://www.ema.europa.eu/docs/en_GB/document_library/Scientific_guideline/2009/09/WC500003904.pdf (accessed March 3, 2020).

